# HERV-W Env Induces Neuron Pyroptosis via the NLRP3–CASP1–GSDMD Pathway in Recent-Onset Schizophrenia

**DOI:** 10.3390/ijms26020520

**Published:** 2025-01-09

**Authors:** Chen Jia, Mengqi Zhang, Xiulin Wu, Xu Zhang, Zhao Lv, Kexin Zhao, Jiahang Zhang, Yaru Su, Fan Zhu

**Affiliations:** 1 State Key Laboratory of Virology and Biosafety, Department of Medical Microbiology, School of Basic Medical Sciences, Wuhan University, Wuhan 430071, China; 2 Hubei Province Key Laboratory of Allergy and Immunology, Wuhan University, Wuhan 430071, China

**Keywords:** HERV-W env, schizophrenia, *IL1B*, *CASP1*, *GSDMD*, *NLRP3*, pyroptosis

## Abstract

HERVs (Human endogenous retroviruses) are remnants of ancient exogenous retroviruses that have integrated into the human genome, particularly in germ-line cells. Among these, the envelope protein gene *HERV-W env* (Human endogenous retroviruses W family envelope protein), located on chromosome 7 and primarily expressed in the human placenta, has been closely linked to various neuropsychiatric disorders, including schizophrenia, as well as autoimmune diseases and cancer. Recent studies have highlighted the abnormal expression of cytokines as a key factor in the pathophysiology of schizophrenia. Notably, elevated serum levels of IL-1β (interleukin 1 beta) in schizophrenia, a cytokine associated with inflammation, are a characteristic feature of pyroptosis—a form of pro-inflammatory programmed cell death. Although previous research has observed significant upregulation of pyroptosis-related genes such as *CASP1* (Caspase-1), *NLRP3* (NLR family pyrin domain containing 3), and *IL1B* (interleukin 1 beta) in the serum of schizophrenia patients, and extensive neuron pyroptosis has been documented in various neuropsychiatric disorders, including Alzheimer’s disease, epilepsy, and multiple sclerosis, the occurrence of neuron pyroptosis in schizophrenia remains uncertain. Furthermore, the mechanisms underlying pyroptosis in schizophrenia and its potential connection with HERV-W env have yet to be fully elucidated. In this study, we found that the expression levels of pyroptosis-related genes, specifically *CASP1*, *GSDMD* (Gasdermin D), and *IL1B*, were significantly elevated in patients with schizophrenia compared to healthy controls. Furthermore, our analysis revealed a strong positive correlation between HERV-W env expression and the levels of *CASP1*/*GSDMD*/*IL1B* in these patients. Experimental evidence further demonstrated that HERV-W env promoted the activation of Caspase-1 and the cleavage of Gasdermin D, leading to increased release of LDH (lactate dehydrogenase) and IL-1β. Importantly, inhibitors targeting *NLRP3*, *CASP1*, and *GSDMD* significantly reduced the releases of LDH and IL-1β induced by HERV-W env, whereas *BID* (BH3 interacting domain death agonist) inhibitors did not have a notable effect. This suggests that HERV-W env induces *CASP1*–*GSDMD*-dependent pyroptosis through the *NLRP3*–*CASP1*–*GSDMD* signaling pathway. As pyroptosis is increasingly recognized for its connection to neurodegenerative diseases, this study provides insights into the molecular mechanisms of neuronal pyroptosis mediated by the NLRP3 inflammasome in the context of HERV-W env. Additionally, it explores the potential facilitation of HERV-W env in the development of schizophrenia via pyroptosis, proposing that certain pyroptosis indicators could serve as potential biomarkers for schizophrenia. Based on our existing research results and the findings of previous researchers, we infer that HERV-W env acts as a bridge in the onset and progression of schizophrenia. Furthermore, HERV-W env may serve as a potential target for the clinical treatment of schizophrenia, suggesting that monoclonal antibody therapy targeting HERV-W env could represent a novel approach to managing this disease.

## 1. Introduction

HERVs (Human endogenous retroviruses) are transposable elements that originated from the integration of ancient retroviral DNA fragments into the human germline genome [1]. Approximately 8% of human genetic material is derived from HERVs, which contain intact retroviral sequences and replicate within the human genome according to Mendelian inheritance [2]. Although most HERVs remain inactive due to deletions and nonsense mutations [3], a select few possess ORFs (open reading frames) capable of encoding functional proteins, contributing to various physiological processes, including embryogenesis [4]. HERVs share the common components of retroviruses, typically comprising gag, pro, pol, and env, along with two long terminal repeats [5]. Based on their relationship with exogenous retroviruses, HERVs are categorized into three major classes: class I (gamma retroviruses and epizootic retroviruses), class II (betaretroviruses), and class III (spumaviruses) [6]. HERVs play important roles in the formation of the placental syncytiotrophoblast layer, antiviral immune defense, and gene transcription [7,8,9,10]. Recent studies showed that pathogens [11], drugs [12], cytokines [13], and epigenetics [14] can activate HERVs, potentially leading to the onset of various diseases, including cancers [15,16] and neuropsychiatric disorders [17,18].

The HERV-W family, which constitutes approximately 1% of the human genome, is classified within the class I. The HERV-W locus on human chromosome 7q21-22 contains a full-length envelope gene, ERVWE1 (Endogenous retrovirus group W member 1, envelope) (or ERVW-1), which codes the HERV-W env (Human endogenous retroviruses W family envelope protein), also known as Syncytin-1 [18]. This protein is expressed in placental syncytiotrophoblasts and is involved in the formation of syncytia during placental morphogenesis [6]. Recent evidence suggests that individuals with schizophrenia exhibit abnormally elevated levels of *HERV-W env* in their blood [17,19,20,21,22,23,24,25,26,27,28,29,30].

Schizophrenia is one of the most devastating and prevalent mental illnesses, affecting about 1% of the global population, and it is characterized by symptoms such as hallucinations, delusions, detachment from reality, and lack of motivation, imposing a significant indirect economic burden on society [31]. While the exact pathogenesis of schizophrenia remains unclear, growing evidence points to an inflammatory response as a contributing factor. Studies show that schizophrenia is associated with elevated levels of pro-inflammatory factors, particularly IL-1β (interleukin 1 beta), IL-6 (interleukin 6), IL-8 (interleukin 8) and TNF-α (tumor necrosis factor alpha-like) [32]. Notably, mature IL-1β is involved in pyroptosis, a form of inflammatory cell death [33].

Pyroptosis is a distinct form of programmed necrosis that involves genes such as *CASP1* (Caspase-1), *GSDMD* (Gasdermin D), *IL1B* (interleukin 1 beta), and inflammasome components like NLRP2 (NLR family pyrin domain containing 2), NLRP3 (NLR family pyrin domain containing 3), NLRC4 (NLR family CARD domain containing 4), and CARD8 (caspase recruitment domain family member 8), primarily operating through the Caspase-1 classical signaling pathway [34]. Caspase-1, [originally termed IL-1β converting enzyme (ICE)], is the first identified caspase responsible for processing pro-IL-1β into its mature form [33]. Gasdermin D is cleaved by Caspase-1, releasing its N-terminal fragment, which mediates pyroptosis and IL-1β secretion [33]. The NLRP3 inflammasome plays a crucial role as a key in activating pyroptosis in health and inflammatory diseases [35]. Pyroptosis is associated with various human diseases, including neurodegenerative diseases, HIV (Human Immunodeficiency Virus)-related neurocognitive disorders, cancers and inflammatory diseases [35,36,37]. Within the context of neurodegenerative diseases and neurocognitive disorders, pyroptosis has gained significant attention in neuroscience and pharmacology [37]. Neuroinflammatory processes are also implicated in the pathophysiology of schizophrenia [38], where microglia in these patients show increased expression of pyroptosis-related genes and reduced responsiveness to IL-1β treatment [38]. However, the potential role of HERV-W env in triggering pyroptosis in neuronal cells in schizophrenia has not been thoroughly explored.

In this study, we analyzed the RNA microarray dataset GSE (Gene Expression Omnibus Series) 25673 from human iPSCs (Induced pluripotent stem cells) and found that differentially expressed genes were primarily involved in pyroptosis and related pathways. Our clinical data showed that pyroptosis-related genes, including *CASP1*, *GSDMD*, and *IL1B*, were significantly elevated in patients with schizophrenia and positively correlated with *HERV-W env* expression. In vitro studies confirmed that HERV-W env enhanced the release of LDH (lactate dehydrogenase) and IL-1β and upregulates *NLRP3*, *CASP1*, *GSDMD*, and *IL1B*, suggesting that HERV-W env promotes pyroptosis via the *NLRP3–CASP1–GSDMD* pathway. These findings indicate that pyroptosis could be a new risk factor for schizophrenia and offer insights into the role of HERV-W env in schizophrenia. This study paves the way for exploring pyroptosis or inflammation-related biomarkers in schizophrenia and highlights the potential of targeting pyroptosis with inhibitors as a treatment strategy.

## 2. Results

### 2.1. Elevated Levels of Expression for Pyroptosis-Associated Genes CASP1, GSDMD, and IL1B in Schizophrenia and Their Correlation with HERV-W Env

**Figure 1:**
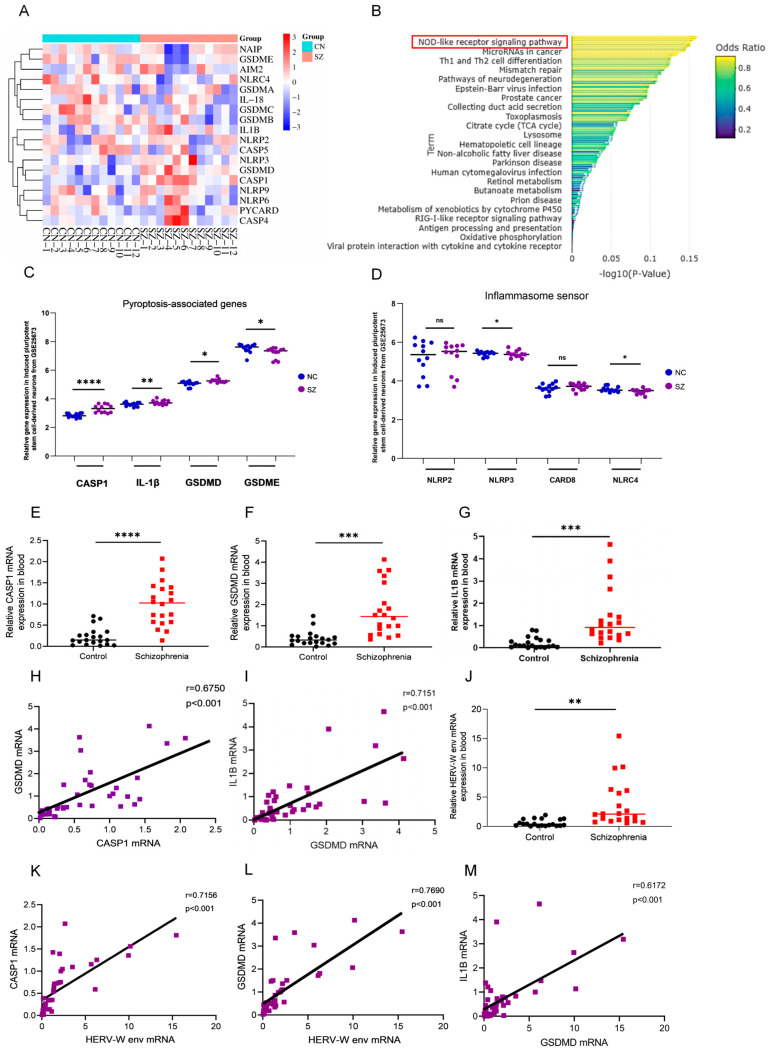
Pyroptosis-associated genes expression and their correlation with HERV-W env in schizophrenia. (**A**) Heat t map showing the expression levels of common pyroptosis-associated genes. (**B**) KEGG analyses of the Top 100 DEGs related to schizophrenia. (**C**) Differential expression of key pyroptosis-associated genes. (**D**) Differential expression of key inflammasome sensor genes. (**E**–**G**). The mRNA levels of *CASP1* (**E**), *GSDMD* (**F**), and *IL1B* (**G**) in healthy controls (*n* = 20) and schizophrenia patients (*n* = 20). (**H**,**I**) Correlation analysis between *GSDMD* with *CASP1* (**H**) and between *GSDMD* and *IL1B* (**I**) expression levels in schizophrenia by Spearman’s analysis. (**J**) The mRNA levels of *HERV-W env* in healthy controls (*n* = 20) and schizophrenia (*n* = 20). (**K**–**M**) Spearman’s correlation analysis between the expression levels of *HERV-W env* and *CASP1* (**K**), *GSDMD* (**L**), and *IL1B* (**M**) in schizophrenia. ns, not significant, * *p* < 0.05, ** *p* < 0.01, *** *p* < 0.001, **** *p* < 0.0001.

GEO (Gene Expression Omnibus) databases are widely used to explore new avenues in disease diagnosis and treatment [39]. In this study, we analyzed the schizophrenia RNA microarray dataset, GSE25673, derived from the iPSCs-derived neurons available in the GEO database. The expression values of pyroptosis-associated genes were illustrated in the heat map and Dot plot (Figure 1A). KEGG (Kyoto Encyclopedia of Genes and Genomes) pathway analysis of the Top 100 DEGs (differentially expressed genes) highlighted the NOD-like (Nucleotide oligomerization domain-like) receptor signaling pathway (Figure 1B). We specifically analyzed the expression of four pyroptosis-associated genes and four inflammasome sensor genes between patients and normal controls. Further analysis of GSE25673 revealed that the levels of *CASP1*, *GSDMD*, *IL1B* (Figure 1C), and *NLRP3* (Figure 1D) were significantly higher in individuals with schizophrenia compared to the control group, while the levels of *GSDME* (Figure 1C) and *NLRC4* (Figure 1D) individuals with schizophrenia were lower than those in the control group, These findings suggest that the expression of the pyroptosis-related genes, particularly *CASP1*, *GSDMD*, *IL1B*, and *NLRP3*, is significantly elevated in neurons derived from iPSCs of schizophrenia patients compared to healthy individuals.

Moreover, our clinical data revealed that the levels of *CASP1*, *GSDMD*, and *IL1B* in the blood of schizophrenia patients were significantly higher than those in healthy controls (Figure 1E–G), consistent with the prediction from bioinformatics analysis of GSE25673. Spearman correlation analysis showed a strong positive correlation between *GSDMD* and *CASP1* (Figure 1H; r = 0.6750) and between *IL1B* and *GSDMD* (Figure 1I; r = 0.7151). Our previous study also indicated elevated HERV-W env levels in schizophrenia patients, and this was further confirmed in the current study, where the mRNA expression in blood samples revealed higher *HERV-W env* levels in schizophrenia patients (Figure 1J). Additionally, Spearman’s analysis demonstrated that *HERV-W env* positively correlated with *CASP1* (Figure 1K; r = 0.7156), *GSDMD* (Figure 1L; r = 0.7690), and *IL1B* (Figure 1M; r = 0.6172) in the blood of schizophrenia patients. These results suggest that the elevated expression of pyroptosis-related genes *CASP1*, *GSDMD* and *IL1B* in schizophrenia is closely associated with *HERV-W env*, making them potential blood biomarkers and pathogenic factors for the disorder.

### 2.2. HERV-W Env Induced Pyroptosis

Clinical studies have shown elevated levels of *CASP1*, *GSDMD*, and *IL1B* in the blood of schizophrenia patients, highlighting their role in the classical pyroptosis pathway [34]. Given the significant association between HERV-W env and schizophrenia, we investigated whether HERV-W env could induce pyroptosis in neuronal cells through cytological experiments.

**Figure 2:**
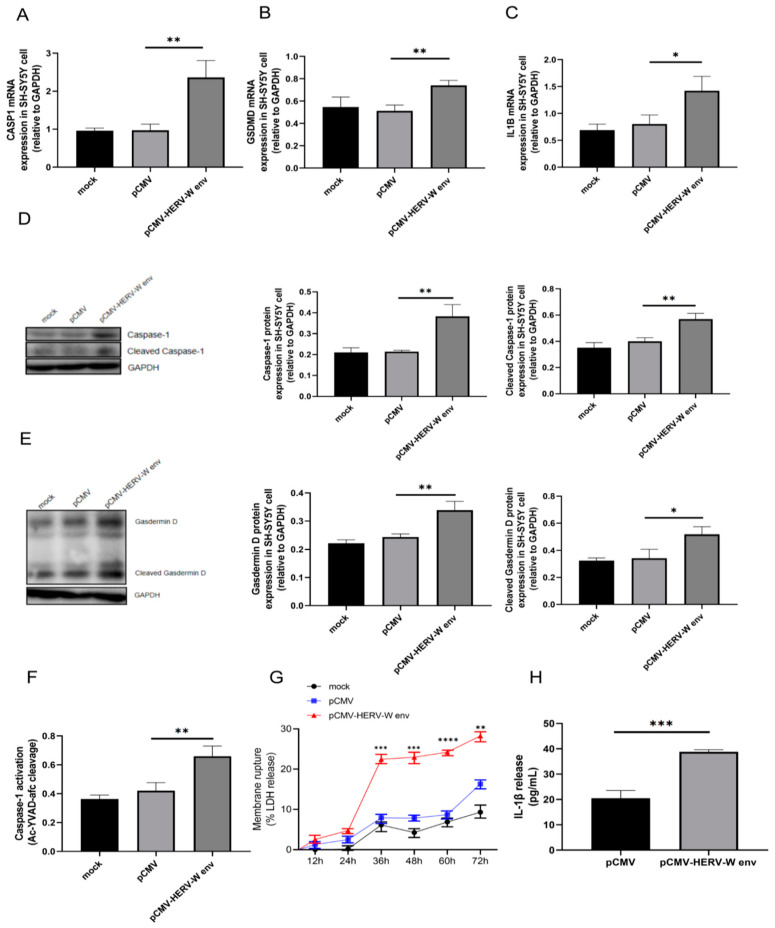
HERV-W env up-regulated the expression of *CASP1*, *GSDMD*, and *IL1B* and promoted the release of LDH and IL-1β. (**A**–**C**) The up-regulation of *CASP1* (**A**), *GSDMD* (**B**), and *IL1B* (**C**) in HERV-W env-transfected SH-SY5Y cells, as detected by RT-qPCR. (**D**–**E**) The up-regulation and cleavage of Caspase-1 (**D**) and Gasdermin D (**E**) in HERV-W env-transfected SH-SY5Y cells, as detected by Western blot. (**F**) Caspase-1 activity in HERV-W env-transfected SH-SY5Y cells, measured using a Multiskan FC plate reader. (**G**) LDH release in HERV-W env-transfected SH-SY5Y cells was measured using the CytoTox 96 LDH Cytotoxicity Assay Kit according to the manufacturer’s protocol. (**H**) IL-1β release in the supernatant of HERV-W env-transfected SH-SY5Y cells, measured by ELISA. Data shown are mean ± SD. * *p* < 0.05, ** *p* < 0.01, *** *p* < 0.001, **** *p* < 0.0001; one-way ANOVA.

We selected SH-SY5Y neuroblastoma cells as a cell model for studying schizophrenia because SH-SY5Y cells are widely used as models for neurodegenerative diseases such as Alzheimer’s disease [40], Parkinson’s disease [41], multiple sclerosis [42] and schizophrenia [43], SH-SY5Y cells exhibit features similar to immature neurons, including the presence of dopamine, GABA (gamma-aminobutyric acid), acetylcholine, and glutamate receptors, which reflects their structural relevance. Furthermore, these cells can replicate specific characteristics of basal ganglia neurons, particularly dopaminergic neurons. When treated with the NMDA (N-methyl-D-aspartic acid receptor) antagonist MK-801, SH-SY5Y cells serve as a model for neuronal damage observed in schizophrenia patients, demonstrating their predictive value. Notably, the tumor origin of SH-SY5Y does not impede their use in studying neuronal phenotypes related to neurodevelopment and neurodegenerative diseases [44,45]. Our results demonstrated that HERV-W env increased mRNA levels of *CASP1* (Figure 2A), *GSDMD* (Figure 2B), and *IL1B* (Figure 2C) following successful transfection, as confirmed by qPCR and Western blot analysis (Supplementary Figure S1). Since the cleavage of Caspase-1 and Gasdermin D is also a hallmark of pyroptosis [36], we found that HERV-W env promoted the cleavage of Caspase-1 (Figure 2D) and Gasdermin D (Figure 2E), with no effect on Gasdermin E protein levels (Supplementary Figure S1). Additionally, HERV-W env promoted the activity of Caspase-1 (Figure 2F) and significantly increased LDH release over time (Figure 2G) in SH-SY5Y cells, along with an approximately 80% increase in IL-1β (Figure 2H) release 48 h after transfection. These findings suggest that HERV-W env enhances pro-inflammatory cell death by upregulating *CASP1*, *GSDMD*, and *IL1B*.

**Figure 3:**
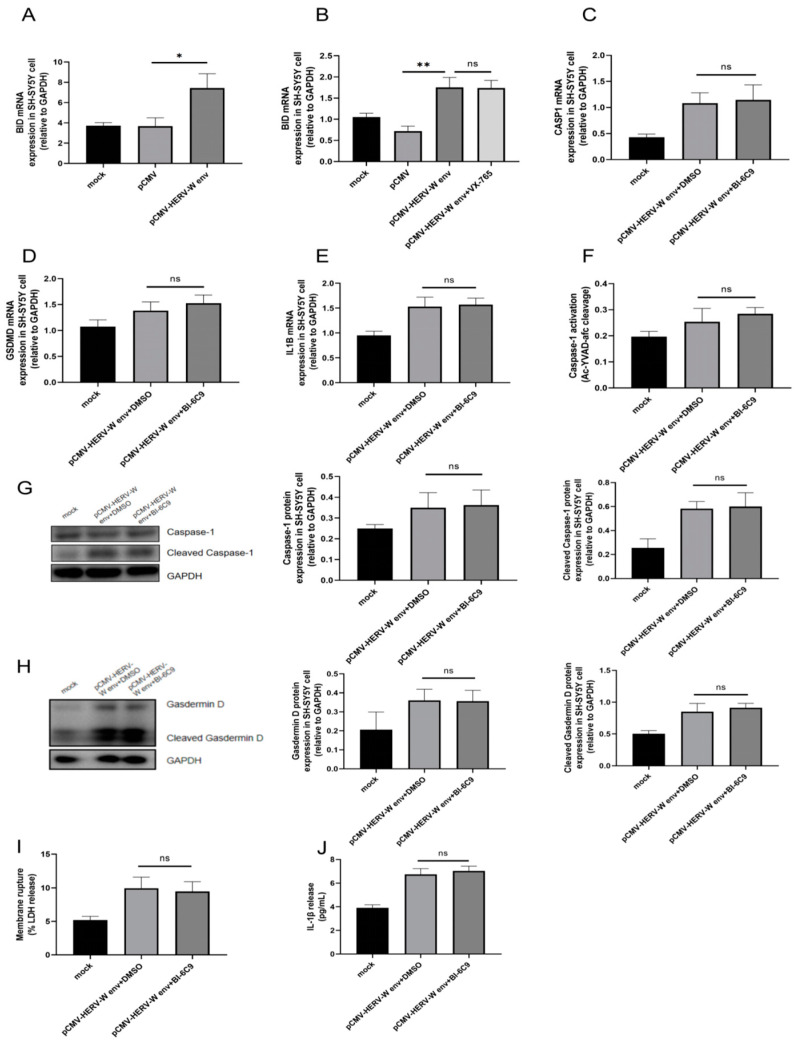
HERV-W env did not induce apoptosis via *BID*. (**A**) The up-regulation of *BID* mRNA in HERV-W env-transfected SH-SY5Y cells, as detected by RT-qPCR. (**B**) Lack of effect of VX-765 on *BID* expression in HERV-W env-transfected SH-SY5Y cells, as detected by RT-qPCR. (**C**–**E**) Lack of effect of BI-6C9 on the expression of *CASP1* (**C**), *GSDMD* (**D**), and *IL1B* (**E**) in HERV-W env-transfected SH-SY5Y cells, as detected by RT-qPCR. (**F**) Lack of effect of BI-6C9 on Caspase-1 activity in HERV-W env-transfected SH-SY5Y cells, measured using a Multiskan FC plate reader. (**G**,**H**) Lack of effect of BI-6C9 on the expression and cleavage of Caspase-1 (**G**) and Gasdermin D (**H**) proteins in HERV-W env-transfected SH-SY5Y cells, as detected by Western blot. (**I**) Lack of effect of BI-6C9 on LDH release in HERV-W env-transfected SH-SY5Y cells, measured using the CytoTox 96 LDH Cytotoxicity Assay Kit according to the manufacturer’s protocol. (**J**) Lack of effect of BI-6C9 on IL-1β release in the supernatant of HERV-W env-transfected SH-SY5Y cells, measured by ELISA. ns, not significant, * *p* < 0.05, ** *p* < 0.01.

However, it is important to note that *CASP1* could influence LDH and IL-1β release through *CASP1-BID* (BH3 interacting domain death agonist) apoptosis pathway other than pyroptosis [46]. Previous studies have shown that Caspase-1 can initiate apoptosis in the absence of GSDMD via a Caspase-1 and Bid-dependent mechanism [46]. We hypothesized that HERV-W env might also affect *BID* expression through *CASP1* upregulation. Our results indicated that HERV-W env increased *BID* mRNA level (Figure 3A), but this effect was not observed when cells were treated with *CASP1* inhibitor VX-765 (Figure 3B). Furthermore, the use of *BID* inhibitor BI-6C9 showed no significant changes in the mRNA level of *CASP1* (Figure 3C), *GSDMD* (Figure 3D) and *IL1B* (Figure 3E), nor Caspase-1 activity (Figure 3F). Protein levels of Caspase-1 (Figure 3G) and Cleaved Caspase-1 (Figure 3G), Gasdermin D (Figure 3H) and Cleaved Gasdermin D (Figure 3H) also remained unchanged. Similarly, LDH (Figure 3I) and IL-1β release (Figure 3J) were unaffected by BI-6C9 treatment. These results suggest that HERV-W env could induce pyroptosis via *CASP1* but not apoptosis.

### 2.3. HERV-W Env Induced Pyroptosis via CASP1 and GSDMD

**Figure 4:**
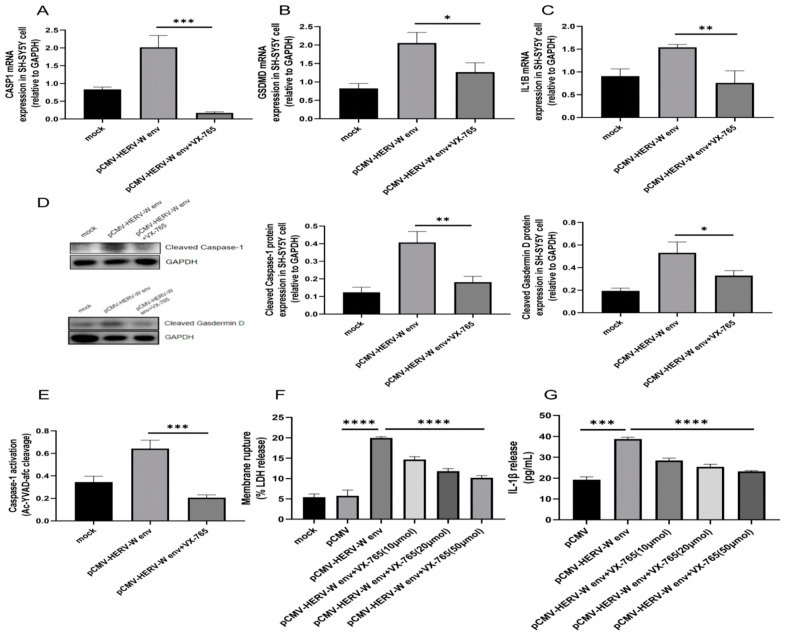
HERV-W env induced pyroptosis via *CASP1*. (**A**–**C**) VX-765 significantly inhibited the expression of *CASP1* (**A**), *GSDMD* (**B**), and *IL1B* (**C**) in HERV-W env-transfected SH-SY5Y cells, as detected by RT-qPCR. (**D**) VX-765 significantly inhibited the cleavage of Caspase-1, Gasdermin D in HERV-W env-transfected SH-SY5Y cells, as detected by Western blot. (**E**) VX-765 significantly reduced Caspase-1 activity in HERV-W env-transfected SH-SY5Y cells, measured using a Multiskan FC plate reader. (**F**) VX-765 significantly inhibited LDH release in a dose-dependent manner in HERV-W env-transfected SH-SY5Y cells, measured using the CytoTox 96 LDH Cytotoxicity Assay Kit according to the manufacturer’s protocol. (**G**) VX-765 significantly inhibited IL-1β release in a dose-dependent manner in the supernatant of HERV-W env-transfected SH-SY5Y cells, as measured by ELISA.* *p* < 0.05, ** *p* < 0.01, *** *p* < 0.001, **** *p* < 0.0001.

Caspase-1 pathway primarily involves the activation of Caspase-1 and the release of LDH, with Gasdermin D acting as the executioner protein [47]. Inflammasome activation triggers *CASP1* through CARD (caspase activation and recruitment domain)-CARD interactions, leading to the cleavage of Gadermin D and IL-1β into their active forms, ultimately resulting in pyroptosis [34]. VX-765, a well-known *CASP1* inhibitor, significantly inhibits Caspase-1-mediated IL-1β production and pyroptosis [48]. Our results indicate that HERV-W env activates a Gasdermin D-related pyroptosis pathway, as the appearance of the Cleaved Gasdermin D segment requires Cleaved Caspase-1. VX-765 treatment significantly reduced the mRNA levels of *CASP1* (Figure 4A), *GSDMD* (Figure 4B), and *IL1B* (Figure 4C), as well as the protein levels of Cleaved Caspase-1 and Cleaved Gasdermin D (Figure 4D), and Caspase-1 activity (Figure 4E). Furthermore, VX-765 inhibited LDH (Figure 4F) and IL-1β release (Figure 4G) in a dose-dependent manner.

**Figure 5:**
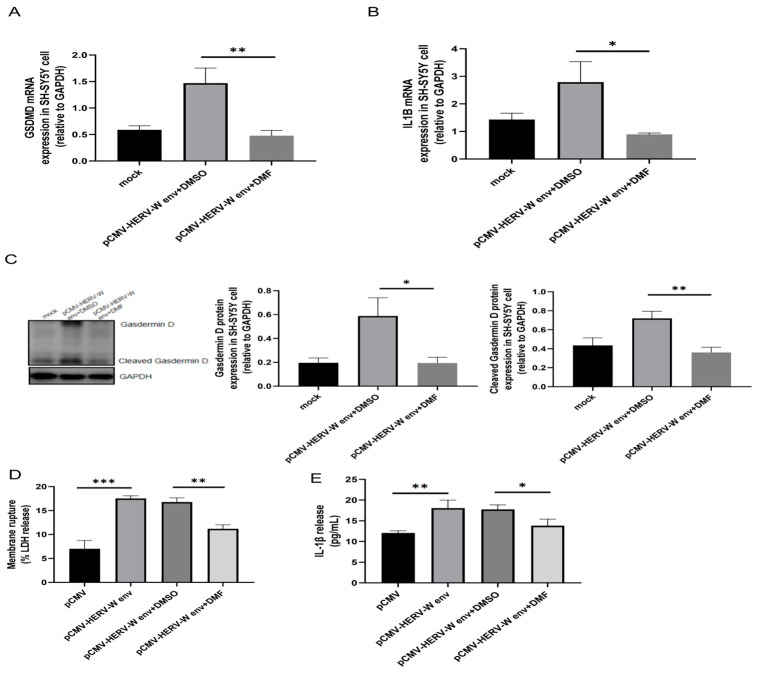
HERV-W env induced pyroptosis via *GSDMD*. (**A**,**B**) DMF significantly reduced the expression of *GSDMD* (**A**) and *IL1B* (**B**) in HERV-W env-transfected SH-SY5Y cells, as detected in RT-qPCR. (**C**) DMF significantly reduced the expression and cleavage of Gasdermin D in HERV-W env-transfected SH-SY5Y cells, as detected by Western blot. (**D**) DMF significantly decreased LDH release in HERV-W env-transfected SH-SY5Y cells, measured using CytoTox 96 LDH Cytotoxicity Assay Kit according to the manufacturer’s protocol. (**E**) DMF significantly reduced IL-1β release in the supernatant of HERV-W env-transfected SH-SY5Y cells, as measured by ELISA. * *p* < 0.05, ** *p* < 0.01, *** *p* < 0.001.

Similarly, DMF (dimethyl fumarate) treatment significantly reduced the mRNA levels of *GSDMD* (Figure 5A) and *IL1B* (Figure 5B), as well as the protein levels of Gasdermin D and Cleaved Gasdermin D (Figure 5C). Furthermore, DMF decreased LDH release (Figure 5D) and IL-1β release (Figure 5E). Importantly, our verification confirmed that DMF’s effect on other antioxidant stress genes, such as NRF2 (nuclear factor erythroid 2-related factor 2), does not interfere with its inhibition of GSDMD or the occurrence of pyroptosis (Supplementary Figure S2). These results indicate that HERV-W env promotes Caspase-1 activation through an upstream pathway, leading to Gasdermin D cleavage, which disrupts the cell membrane and causes the release of LDH and IL-1β. We conclude that HERV-W env activates the Caspase-1-dependent pyroptosis pathway involving Cleaved Gasdermin D in cell membrane rupture, independently of *BID*. We propose that inflammasomes, key cytoplasmic protein complex response to PAMPs (pathogen-associated molecular patterns) and DAMPs (damage-associated molecular patterns), are crucial upstream activators of this pyroptosis pathway.

### 2.4. HERV-W Env Initiated the Pyroptosis Pathway via the NLRP3 Inflammasome Sensor

**Figure 6:**
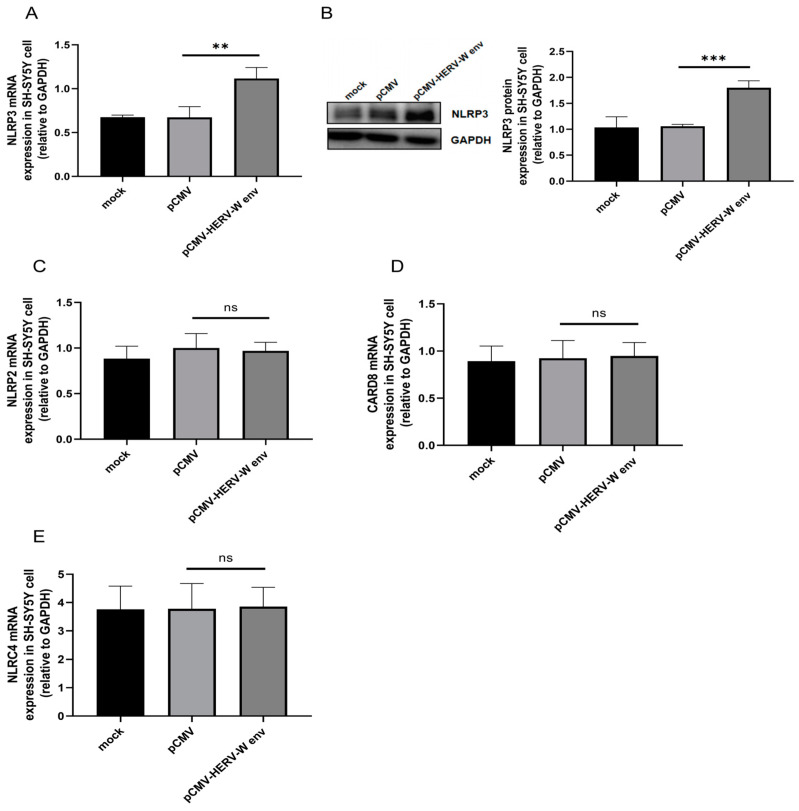
HERV-W env promoted NLRP3 inflammasome sensor. (**A**) Upregulation of *NLRP3* mRNA in HERV-W env-transfected SH-SY5Y cells, as detected by RT-qPCR. (**B**) Upregulation of NLRP3 protein in HERV-W env-transfected SH-SY5Y cells, as detected by Western blot analysis. (**C**–**E**) HERV-W env did not affect the expression of *NLRP2* (**C**), *CARD8* (**D**), and *NLRC4* (**E**) in SH-SY5Y cells, as detected by RT-qPCR. ns, not significant, ** *p* < 0.01, *** *p* < 0.001.

Inflammasomes are cytosolic protein complexes essential for innate immunity, particularly in myeloid cells [49]. Activation of inflammasomes is triggered by the binding of cytoplasmic sensor proteins to microbial-associated molecular patterns or DAMPs produced by stressed or dying host cells [49]. Bioinformatics previously suggested a link between schizophrenia and *NLRP3*. We found that HERV-W env increased *NLRP3* mRNA (Figure 6A) and protein levels (Figure 6B) with no effect on the mRNA levels of *NLRP2* (Figure 6C), *CARD8* (Figure 6D) or *NLRC4* (Figure 6E). These results suggest that HERV-W env likely activates inflammasomes by upregulating *NLRP3* expression, thereby mediating the Caspase-1-dependent pyroptosis pathway.

**Figure 7:**
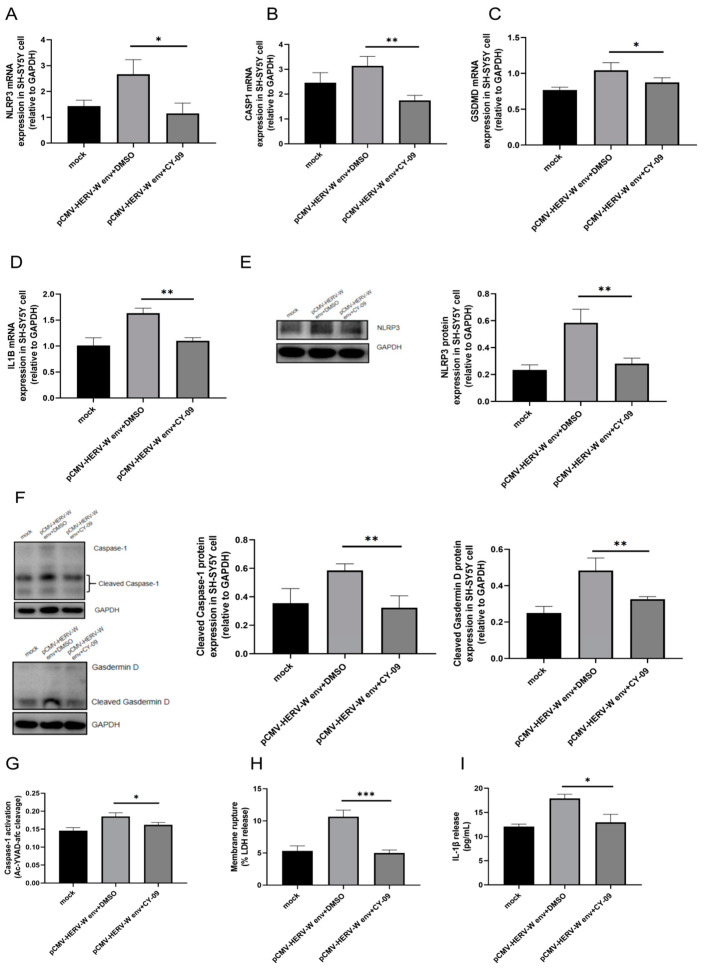
HERV-W env induced pyroptosis via *NLRP3*. (**A**–**D**) CY-09 reduced the expression of *NLRP3* (**A**), *CASP1* (**B**), *GSDMD* (**C**), and *IL1B* (**D**) in HERV-W env-transfected SH-SY5Y cells, as detected by RT-qPCR. (**E**,**F**) CY-09 reduced the expression of NLRP3 (**E**) and the cleavage of Caspase-1 and Gasdermin D (**F**) in HERV-W env-transfected SH-SY5Y cells, as detected by Western blot. (**G**) CY-09 reduced Caspase-1 activity in HERV-W env-transfected SH-SY5Y cells, measured using a Multiskan FC plate reader. (**H**) CY-09 reduced LDH release in HERV-W env-transfected SH-SY5Y cells, measured using the CytoTox 96 LDH Cytotoxicity Assay Kit according to the manufacturer’s protocol. (**I**) CY-09 reduced IL-1β release in the supernatant of HERV-W env-transfected SH-SY5Y cells, as measured by ELISA. Statistical analysis was performed by one-way analysis of variance (ANOVA). * *p* < 0.05; ** *p* < 0.01., *** *p* < 0.001.

To confirm whether HERV-W env initiates pyroptosis through the NLRP3 inflammasome sensor, we utilized *NLRP3* specificity inhibitor CY-09 (MedChemExpress, HY-103666). Treatment with 1 μmol CY-09 significantly reduced the mRNA levels of *NLRP3* (Figure 7A), *CASP1* (Figure 7B), *GSDMD* (Figure 7C) and *IL1B* (Figure 7D). Protein levels of NLRP3 (Figure 7E), Caspase-1 (Figure 7F) and Cleaved Gasdermin D (Figure 7F) were also significantly decreased. Caspase-1 activity (Figure 7G), LDH release (Figure 7H) and IL-1β release (Figure 7I) were similarly inhibited by CY-09 treatment. Overall, our findings suggest that HERV-W env ultimately leads to pyroptosis through the *NLRP3*–*CASP1*–*GSDMD* pathway, resulting in the release of large amounts of LDH and IL-1β.

## 3. Discussion

HERVs have emerged as a novel pathogenic factor [50]. Based on existing research, the pathogenic mechanisms of HERVs can be summarized in several key aspects: (1) the presence of a helper virus can lead to the formation of complete virus-like particles, resulting in infection and disease [19]; (2) integration of HERVs LTRs (long terminal repeats) into the host oncogenes can cause cancers [51]; (3) the expression of viral proteins in host cells can alter signaling pathways, ultimately leading to disease [52]. Retrovirus has been speculated as one of the potential infectious agents in the development of schizophrenia [53,54]. A recent meta-analysis [55] provided valuable insights. This study analyzed 13 eligible studies, comprising 698 cases and 728 controls from various regions worldwide. The analysis included samples such as serum, plasma, PBMCs and leukocytes, CSF, and postmortem brain tissues for the detection of HERV-W. In the subgroup analysis, among the various HERV-W fragments, the elevated expression of HERV-W envelope protein or RNA in blood demonstrated the strongest association with schizophrenia. In 2011, we proposed the hypothesis that HERV-W env plays a pathogenic role in schizophrenia, with both genetic and environmental factors contributing to its pathogenesis. HERVs serve as a bridge and a trigger, where external, internal, or epigenetic factors activate HERVs, initiating the disease onset [17]. Our subsequent studies have provided evidence supporting this hypothesis. It has been shown that HERV-W env influences schizophrenia pathogenesis through several mechanisms. For instance, HERV-W env can lead to alterations in protein modifications, modulating the expression of schizophrenia-related genes [56]. It can induce organelle abnormalities, such as inducing neuronal mitochondrial dysfunction via activating AK2 (adenylate kinase 2) [21], suppression of mitochondrial complex I activity via down-regulation of CPEB1 (Cytoplasmic Polyadenylation Element Binding Protein 1) and NDUFV2 (NADH: Ubiquinone Oxidoreductase Core Subunit V2) [26] and ER (endoplasmic reticulum) stress caused by decreasing GANAB (Glucosidase II Alpha Subunit) levels [27]. HERV-W env also affects neuronal ion channels [24,57,58], leading to potential disruptions in neuronal activity [28], and causes morphological changes in neurons [29], affecting neuronal plasticity [25]. Furthermore, it induces inflammation by increasing iNOS (inducible nitric oxide synthase) [59], stimulating the production of cytokines such as TNF-α [60], IL-10 (interleukin 10) [60], CRP (C-reactive protein) [23], IL-6 [23], and IFN-β (interferon-β) [20], or eliciting CTL (cytotoxic T lymphocyte) responses [61]. It can even trigger programmed cell death, including apoptosis [20] and ferroptosis [30]. In this study, we discovered that HERV-W env promotes pyroptosis by increasing the expression of *NLRP3*, *CASP1*, *GSDMD* and *IL1B*, potentially representing a novel mechanism by which HERV-W env influences neuronal cell death in patients with schizophrenia.

The course and pathophysiology of schizophrenia are complex and influenced by numerous environmental and epigenetic factors [62]. For example, the lack of environmental resources may exacerbate negative symptoms in patients with schizophrenia [63]. Furthermore, by reconstructing the regulatory process of cell type development and differentiation, the researchers have identified that common variations associated with schizophrenia strongly overlap with cell type-specific regulatory regions connected by the chromatin loop [64], and these are making accurate diagnosis and treatment challenging [65]. For a long time, researchers have actively explored the causal relationship between blood-based biomarkers and schizophrenia. Studies have shown that certain blood-based biomarkers, such as CRP [66] and CD33 [67], are causally linked to various psychiatric disorders, including schizophrenia, Alzheimer’s disease, and Parkinson’s disease. Recent studies have demonstrated the potential of using a cell model to study brain markers associated with schizophrenia. For example, iPSCs can be derived into brain organoids [68] to represent gene expression in brain tissue. To simulate the neuropathology of schizophrenia during the early critical period of brain development, scientists have generated 3D brain organoids using patient-derived iPSCs. These studies revealed that endothelial cells in schizophrenia brain organoids exhibit higher permeability and altered patterns of angiogenesis. Furthermore, iPSCs can differentiate into trophoblast stem cells, which are similar to those derived from human blastocysts or first-trimester placentas and can express brain-related genes [69]. These findings provide important clues for understanding the pathogenesis of schizophrenia and identifying potential therapeutic targets. Therefore, understanding the specific mechanisms and identifying biomarkers of schizophrenia are critical. IPSCs are pluripotent stem cells derived from somatic cells through reprogramming techniques, with the potential to differentiate into multiple cell types, including brain cells. By obtaining somatic cells from a patient’s body and reprogramming them into iPSCs, researchers can generate brain cells with the same genetic background as the patient. This approach enables the study of brain disease pathogenesis and the screening of potential treatments. For example, in schizophrenia research, patient-derived iPSCs have been used to generate 3D brain organoids. These studies revealed a higher proportion of endothelial cells in schizophrenia brain organoids, accompanied by increased expression of related genes [68]. This discovery offers a novel perspective on understanding the pathophysiology of schizophrenia. Moreover, changes in plasma marker levels are also closely related to the occurrence and progression of brain diseases. For instance, in patients with schizophrenia, plasma levels of certain inflammatory factors may fluctuate [32], potentially reflecting immune and inflammatory responses in the brain. Gene expression patterns in the brain can influence the levels of specific biomolecules in plasma. Certain brain genes may encode proteins or peptides secreted into the plasma, serving as plasma markers. Additionally, brain gene expression may affect hormones and other biomolecule levels in plasma by regulating the neuroendocrine system [70,71]. Through bioinformatics analysis of the GSE25673 dataset from the GEO database [72], we identified widespread inflammation in patients with schizophrenia, with DEGs associated with the NOD-like receptor signaling pathway and other pathways linked to Alzheimer’s disease, glycerolipid metabolism, and Kaposi’s sarcoma-associated herpesvirus infection, all of which involve pyroptosis. These findings suggest that pyroptosis may play a role in the pathophysiology of schizophrenia.

Pyroptosis, an inflammatory form of programmed cell death, was first identified in response to invasive pathogenic bacteria in 1992 [73]. Since then, it has been linked to the progression of various human diseases, including neurodegenerative disorders, HIV-associated neurocognitive disorders, cancers, and cardiovascular diseases [36]. Previous studies have indicated a significant correlation between pyroptosis and neuropsychiatric disorders, such as Alzheimer’s disease, epilepsy, and multiple sclerosis [74,75,76]. Certain viral infections, including SARS-CoV-2 (severe acute respiratory syndrome coronavirus 2) [77] and enterovirus 71 [78], can also trigger pyroptosis. Previous research has observed significant upregulation of pyroptosis-related genes, such as *CASP1* [79], *NLRP3* [80], and *IL1B* [32], in the serum of schizophrenia patients. However, whether neuron pyroptosis occurs in schizophrenia and the molecular mechanisms linking pyroptosis and schizophrenia remain insufficiently explored. The mechanisms underlying pyroptosis in neurological conditions primarily involve the activation of the inflammasome, Caspase-1 activation, Gasdermin-D cleavage, and the subsequent release of LDH and IL-1β. Previous studies have shown that HERV-W env has immunomodulatory properties within the nervous system [59,60,61], inducing the expression of pro-inflammatory cytokines like interleukin IL-1β in PBMCs (peripheral blood mononuclear cells) [81]. Our clinical findings indicate a strong association between HERV-W env and the pyroptosis-related genes *CASP1*, *GSDMD*, and *IL1B* in individuals with schizophrenia. Consequently, we investigated the impact of HERV-W env on pyroptosis in cytological experiments and found that HERV-W env induces pyroptosis, enhancing the release of LDH and IL-1β.

The molecular signaling pathways governing pyroptosis are intricate, with Caspase-1 and Gasdermin D playing central roles [34]. Pyroptosis differs from apoptosis by selectively releasing specific cytoplasmic contents, including pro-inflammatory cytokines and DAMPs [82]. While *BID* can mediate apoptosis through *CASP1*, our results show that inhibiting *BID* does not significantly affect the pyroptosis induced by HERV-W env, suggesting that HERV-W env induces pyroptosis via *CASP1* rather than apoptosis. Among the GSDM (Gasdermin) family, Gasdermin D is the key executor of inflammasome-induced pyroptosis. In the CNS (Central Nervous System), *GSDMD* is expressed under physiological conditions and is upregulated during CNS diseases or injuries [83]. Due to the necessity of for further research into specific small molecule inhibitors targeting *GSDMD* for large-scale application, we selected DMF as the *GSDMD* inhibitor. Previous studies have shown that DMF effectively blocks pyroptosis in the Gasdermin D pathway without being significantly affected by DMF’s interactions with other targets [84]. DMF’s mechanism involves activation of the NRF2 pathway [85], along with targeting GAPDH and autoimmune glycolysis to modulate immunity [86]. Our results indicated that both the *CASP1* inhibitor VX-765 and *GSDMD* inhibitor DMF significantly suppressed pyroptosis induced by HERV-W env, supporting the conclusion that HERV-W env induces cell pyroptosis via *CASP1*–*GSDMD*.

The inflammasome complex undergoes autocatalytic cleavage, producing two active Cleaved-Caspase-1 subunits, p10 and p20 [87]. Within the pyroptosis pathway, the NLR-containing protein family plays a critical role as activators. This family is structurally characterized by a central NBD (nucleotide-binding domain), a C-terminal leucine-rich repeat domain, and an N-terminal domain [49]. The NLR protein family is divided into subfamilies based on their N-terminal domains: NLRP (pyrin) and NLRC (CARD). While members like *NLRP1* (NLR family pyrin domain containing 1), *NLRP3*, *NLRC4*, and *NLRP6* (NLR family pyrin domain containing 6) are well-established inflammasome sensors, others, like *NLRP12* (NLR family pyrin domain containing 12), are still under investigation [87]. Our findings showed that HERV-W env significantly upregulated *NLRP3* mRNA and protein levels without affecting *NLRP2*, *CARD8*, and *NLRC4* mRNA. This suggests that HERV-W env may induce pyroptosis by activating the NLRP3 inflammasome. Among the NLR family, the NLRP3 inflammasome is one of the most extensively studied inflammasomes and has been implicated in various CNS diseases [88]. NLRP3′s unique ability to recognize a wide range of activators, including microbial components, environmental crystalline pollutants, and endogenous danger signals, underlies its involvement in these diseases [89]. In the brain, *NLRP3* is expressed in multiple cell types, including microglia, astrocytes, and neurons [87,90,91]. It is particularly noteworthy that in schizophrenia, iPSC-astrocytes exhibit unique NLRP3-dependent inflammatory characteristics, which are manifested through various cellular functions. Caspase-1 activity was found to increase significantly following specific NLRP3 stimulation compared with the control group [92]. Silencing *NLRP3* through siRNA has been shown to enhance the inhibitory effects of curcumin on microglial pyroptosis and proinflammatory responses, highlighting the therapeutic potential of targeting *NLRP3* [91]. Recent studies have also demonstrated that the NLRP3 inflammasome plays a role in HIV gp120 protein-induced pyroptosis in microglia [36] and in SARS-CoV-2-induced pyroptosis [93]. Similarly, our findings discovered that HERV-W env could induce NLRP3-dependent pyroptosis in SH-SY5Y, and inhibition of *NLRP3* with CY-09 significantly reduces the release of LDH and IL-1β. These findings underscore the potential of targeting the NLRP3 inflammasome in conditions where HERV-W env is implicated. Importantly, recent data suggest that SARS-CoV-2 can induce the expression of HERV-W env protein and participate in the immunopathogenesis of certain COVID-19 (Corona Virus Disease 2019) related syndromes [94]. This raises the possibility that SARS-CoV-2 may enhance NLRP3-dependent pyroptosis through the upregulation of HERV-W env, implying that HERV-W env could serve as a potential therapeutic target for both schizophrenia and inflammatory respiratory diseases caused by SARS-CoV-2.

GNBAC1, an IgG4 monoclonal antibody that specifically targets HERV-W-env, has shown promise in clinical treatment for multiple sclerosis [95] and is currently being developed for T1D (type 1 diabetes) [96]. Our findings suggest that HERV-W env could also be a potential target for the clinical treatment of schizophrenia, indicating that monoclonal antibody therapy targeting HERV-W-env may represent a novel approach to managing this disorder.

## 4. Materials and Methods

### 4.1. Bioinformatics

We obtained the gene expression profile of induced pluripotent stem cell-derived neurons from the GSE25673 on the GPL6244 platform (Affymetrix Human Gene 1.0 ST Array), which includes data from both schizophrenic and normal individuals. Microarray data were downloaded using the GEO query. DEGs were visualized with heat maps using online bioinformatics tools. (http://www.bioinformatics.com.cn, accessed on 30 June 2023) Additionally, we use GEO Explorer to visualize the enrichment of the top 100 DEGs in KEGG pathways and GO (gene ontology) biology biological processes (http://geoexplorer.rosalind.kcl.ac.uk, accessed on 3 July 2023).

### 4.2. Clinical Samples and Ethical Considerations

Blood samples from patients with schizophrenia and a healthy control group were obtained from the Renmin Hospital, Wuhan University. All patients met the criteria for recent onset schizophrenia as defined in the fifth edition of the Diagnostic and Statistical Manual of Mental Disorders. The 20 patients included had no history of antipsychotic drug treatment and did not exhibit symptoms of acute infectious, inflammatory, or neurological diseases. The control group consisted of 20 healthy donors. Informed consent was obtained from all participants, and the study was conducted in compliance with ethical principles. Plasma samples were treated with ethylenediamine tetraacetic acid (EDTA) and immediately stored at −80 °C until analysis. There were no significant differences in median age, education level, body mass index, smoking status, and gender distribution between the schizophrenia patients and the control group. Demographic information from the cohort of subjects in the plasma level study were listed in Supplementary Table S3.

### 4.3. Plasmid Construction

The previously obtained HERV-W env sequence [17] was cloned into the mammalian expression vector pCMV.

### 4.4. Cell Culture and Transfection

The neuroblastoma cell line SH-SY5Y was purchased from the American Type Culture Collection. Cells were cultured in a 1:1 mixture of Minimal Essential Medium Eagle (MEM) (2225320, Gibco, Baltimore, MD, USA) and F-12 (2209586, Gibco, Baltimore, MD, USA), supplemented with 10% fetal bovine serum (2001003, Biological Industries, Beit HaEmek, Israel), 100mmol/L sodium pyruvate (2185865, Gibco, Baltimore, MD, USA) and 1% penicillin/streptomycin (15140148, Gibco, Baltimore, MD, USA). The cells were maintained at 37 °C with 5% CO_2_. Transfection was performed using the NeofectTM DNA Transfection reagent (D210101, Neofect Biotech Co., Ltd., Beijing, China) according to the manufacturer’s instructions.

### 4.5. ELISA Assay

The expression level of IL-1β in culture supernatants was determined using an ELISA kit (ABclonal, RK00001, Wuhan, China) according to the manufacturer’s instructions. Absorbance was measured at 450 nm using a Multiskan FC plate reader (Thermo Fisher Scientific, 357-900694, Beijing, China).

### 4.6. Inhibitor Treatment

Inhibitors were added to cells 24–48 h after plasmid transfection, either at a continuous dilution or a specific concentration. The following inhibitors were used: Belnacasan (VX-765) (MedChemExpress, HY-13205, Monmouth Junction, NJ, USA) at 50 μmol. Dimethyl fumarate (DMF) (MedChemExpress, HY-17363, Monmouth Junction, NJ, USA) at 5 μmol in dimethyl sulfoxide (DMSO). CY-09 (MedChemExpress, HY-103666, Monmouth Junction, NJ, USA) at 1 μmol in DMSO. ML385 (MedChemExpress, HY-100523, Monmouth Junction, NJ, USA) at 5 μmol in DMSO. Heptelidic acid (HA) (MedChemExpress, HY-100523, Monmouth Junction, NJ, USA) at 1 μmol in DMSO.

### 4.7. Analysis of mRNA Expression

Total RNA was isolated from cultured cells using TRIzol reagent (Invitrogen, Carlsbad, CA, USA). Genomic DNA contamination was removed using DNase I (Thermo Fisher Scientific, EN0521, Waltham, MA, USA) according to the manufacturer’s recommendation. Reverse transcription was performed with the MMLV Reverse Transcriptase cDNA Synthesis Kit (Invitrogen, 18091200, Carlsbad, CA, USA). Real-time Quantitative Polymerase Chain Reaction (RT-qPCR) was conducted on an iCycler system^®^ (Bio-Rad, Hercules, CA, USA) using SYBR Select Master Mix (Invitrogen, Carlsbad, CA, USA). GAPDH or β-actin was used as the housekeeping gene for normalization. Primers were designed using sequences from the NCBI database with Primer 5.0 software, as listed in Supplementary Table S1.

### 4.8. Western Blot Analysis

Forty-eight hours after transfection, cells were washed with phosphate-buffered saline (PBS) and lysed by M-PER reagents (78501, Pierce Chemical, Rockford, IL, USA) containing protein inhibitors (ab201119, Abcam, Cambridge, UK). Protein concentration was determined using the Pierce TM BCA Protein Assay (UD281372; Thermo Fisher Scientific, Waltham, MA, USA). Samples were loaded onto a 10% SDS-PAGE, followed by electrotransfer to a PVDF membrane (IPVH00010; Amersham Biosciences, Piscataway, NJ, USA). Membranes were incubated with primary antibodies overnight at 4 °C and then with secondary antibodies at room temperature for one hour. Protein bands were visualized using an ECL chemiluminescence solution (SW2030, Biosharp, Hefei, China) and an automatic chemiluminescence system (5200, Tanon, Shanghai, China). Relative protein expression levels were qualified to GAPDH. Antibodies used in this study were listed in Supplementary Table S2.

### 4.9. LDH Release Assay

LDH release into culture supernatants was measured using the CytoTox 96 LDH Cytotoxicity Assay Kit (Beyotime, C0017, Shanghai, China) according to the manufacturer’s protocol. The percentage of LDH release was calculated as follows: % LDH release = (sample LDH activity − background LDH)/(total LDH activity − background LDH) × 100.

### 4.10. Caspase-1 Activity Assay

Caspase-1 activity was detected by measuring the cleavage of the substrate Ac-YVAD-AFC (MedChemExpress, HY-P2377, Monmouth Junction, NJ, USA), which produces a fluorescent signal. Absorbance was measured at 380 nm using a Multiskan FC plate reader (Thermo Fisher Scientific, 357-900694, Beijing, China).

### 4.11. Statistical Analysis

Statistical analysis was performed using SPSS13.0 and GraphPad Prism8.0. Median analysis and Mann–Whitney U analysis were used to compare the differential expression of *HERV-W env*, *CASP1*, *GSDMD*, and *IL1B* between schizophrenia patients and controls. Spearman rank correlation was used for correlation analysis. The signal pathway diagram was created using Adobe Illustrator CC 2018. Data were obtained from at least three independent experiments. One-way ANOVA and Student’s *t*-test were used for other statistical analyses. A *p*-value of <0.05 was considered statistically significant.

## 5. Conclusions

**Figure 8:**
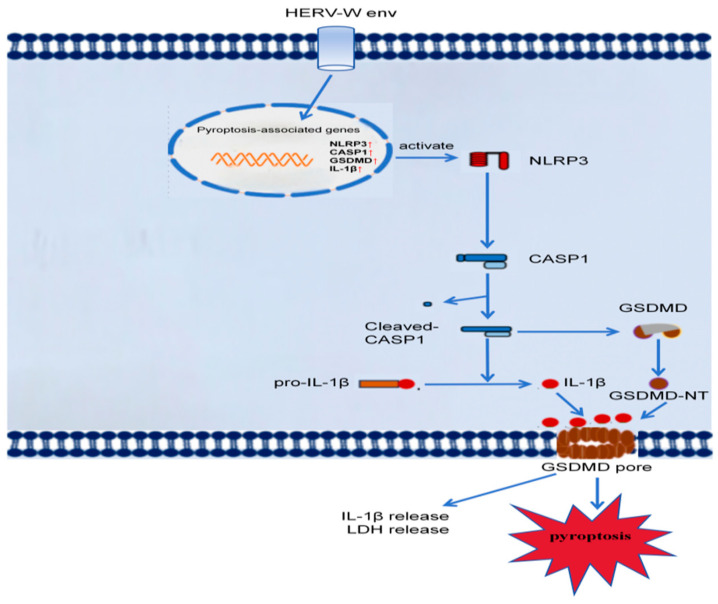
HERV-W env induced pyroptosis via NLRP3-CASP1-GSDMD pathway. HERV-W env promoted the expression of *NLRP3*, *CASP1*, *GSDMD*, and *IL1B* (as indicated by the red up arrows), along with the cleavage of Caspase-1 and Gasdermin D. This process leads to the release of LDH and IL-1β, which are markers of pyroptosis.

Our clinical observations revealed that the levels of *CASP1*, *GSDMD*, and *IL1B* were significantly elevated in the blood of patients with schizophrenia and positively correlated with *HERV-W env*. Further investigation showed that HERV-W env upregulated the expression of *NLRP3*, *CASP1*, and *GSDMD*, which in turn promoted the release of LDH and IL-1β, ultimately inducing pyroptosis (Figure 8). These findings suggest a novel mechanism by which HERV-W env induces *CASP1*–*GSDMD*-dependent neuron pyroptosis through the *NLRP3*–*CASP1*–*GSDMD* signaling pathway, providing new insights into the potential pyroptosis mechanisms by which HERV-W env contributes to the onset and progression of schizophrenia.

## Data Availability

All data generated or analyzed in this study are included in this article.

## Supplementary Materials

The supporting information can be downloaded at: https://www.mdpi.com/article/10.3390/ijms26020520/s1.

## Abbreviations

HERVs, Human endogenous retroviruses; HERV-W env, Human endogenous retroviruses W family envelope protein; ERVWE1, Endogenous retrovirus group W member 1, envelope; CASP1, Caspase-1; GSDMD, Gasdermin D; GSDME, Gasdermin E; IL1B (IL-1β), interleukin 1 beta; BID (Bid), BH3 interacting domain death agonist; NLRP2; NLR family pyrin domain containing 2; NLRP3, NLR family pyrin domain containing 3; NLRC4, NLR family CARD domain containing 4; CARD8, caspase recruitment domain family member 8; IL-6, interleukin 6; IL-8, interleukin 8; TNF-α, tumor necrosis factor alpha-like; CARD, caspase activation and recruitment domain; LDH, lactate dehydrogenase; NRF2, nuclear factor erythroid 2-related factor 2; HIV, Human immunodeficiency virus; GSE, Gene Expression Omnibus Series; iPSCs, Induced pluripotent stem cells; GEO, Gene Expression Omnibus; NAIP, NLR family apoptosis inhibitory protein; AIM2, absent in melanoma 2; GSDMA, Gasdermin A; IL-18, interleukin 18; GSDMB, Gasdermin B; GSDMC, Gasdermin C; CASP5, Caspase-5; NLRP9, NLR family pyrin domain containing 9; PYCARD, PYD and CARD domain containing; CASP4, Caspase-4; KEGG, Kyoto Encyclopedia of Genes and Genomes; DEGs, differentially expressed genes; NOD-like, Nucleotide oligomerization domain-like; GABA, gamma-aminobutyric acid; NMDA, N-methyl-D-aspartic acid receptor; PAMPs, pathogen-associated molecular patterns; DAMPs, damage-associated molecular patterns; ORFs, open reading frames; LTRs, long terminal repeats; DMF, dimethyl fumarate; AK2, adenylate kinase 2; CPEB1, Cytoplasmic Polyadenylation Element Binding Protein 1; NDUFV2, NADH: Ubiquinone Oxidoreductase Core Subunit V2; ER, endoplasmic reticulum; GANAB, Glucosidase II Alpha Subunit; iNOS, inducible nitric oxide synthase; IL-10, interleukin 10; CRP, C-reactive protein; IFN-β, interferon-β; CTL, cytotoxic T lymphocyte; SARS-CoV-2, severe acute respiratory syndrome coronavirus 2; PBMCs, peripheral blood mononuclear cells; GSDM, Gasdermin; CNS, Central Nervous System; NBD, nucleotide-binding domain; NLRP1, NLR family pyrin domain containing 1; NLRP6, NLR family pyrin domain containing 6; NLRP12, NLR family pyrin domain containing 12; COVID-19, Corona Virus Disease 2019; T1D, type 1 diabetes; GO, gene ontology.
