# Supplementary material for: HERV-W Env Induces Neuron Pyroptosis via the NLRP3–CASP1–GSDMD Pathway in Recent-Onset Schizophrenia
Source: Int J Mol Sci. 2025 Jan 9;26(2):520. doi: 10.3390/ijms26020520 (PMC11765033; doi:10.3390/ijms26020520)
Supplement: Supplementary file 1 [file ijms-26-00520-s001.zip › Supplementary Figure S1.pdf]

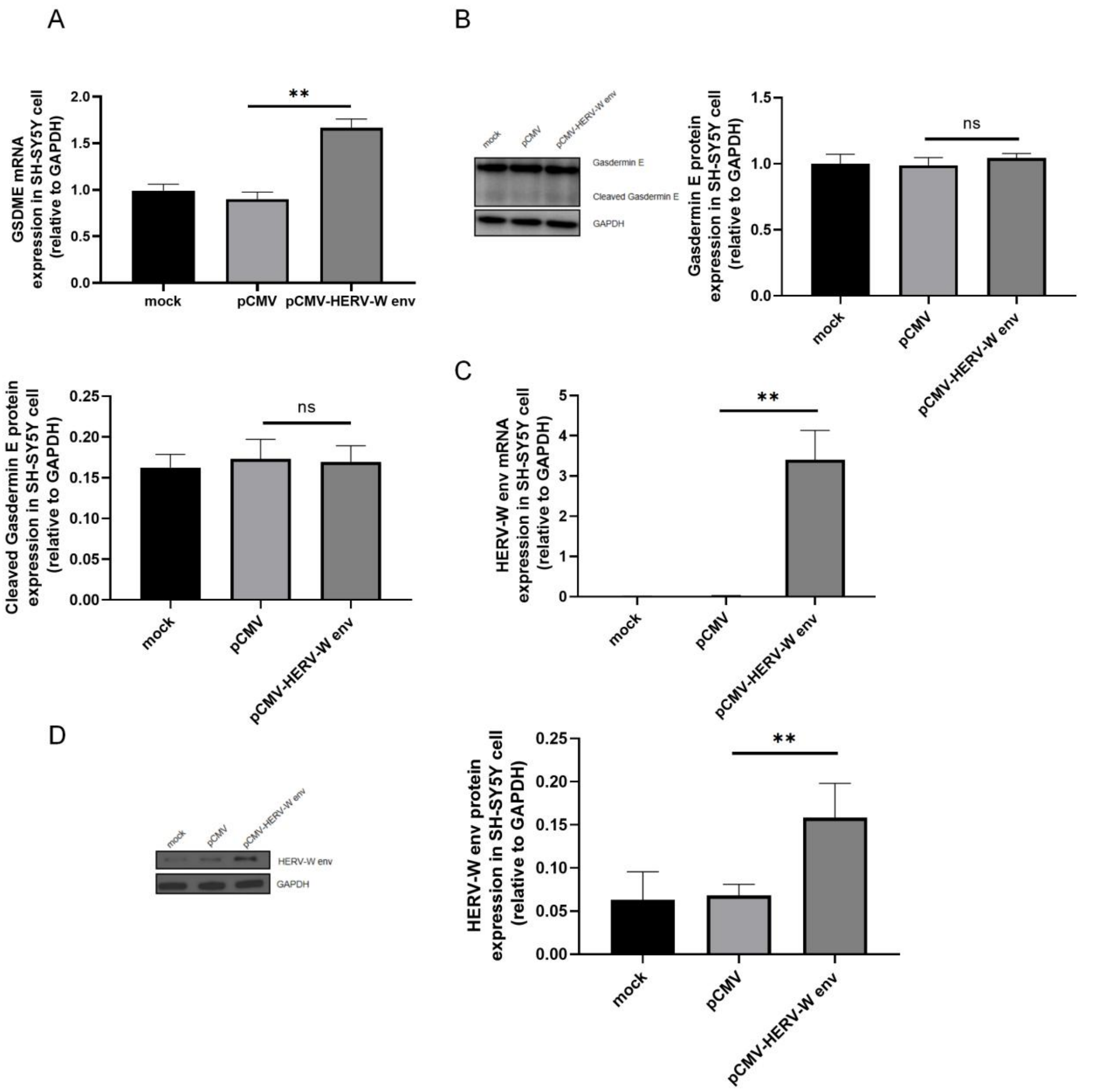

Supplementary Figure S1. The successful transfection of HERV-W env and HERV-W env had no effect on the protein levels of Gasdermin E. (A). Upregulation of *GSDME* mRNA in HERV-W env-transfected SH-SY5Y cells, as detected by RT-qPCR. (B). HERV-W env had no effect on the protein levels of Gasdermin E in HERV-W env-transfected SH-SY5Y cells, as detected by Western blot analysis. (C). Upregulation of *HERV-W env* mRNA in HERV-W env-transfected SH-SY5Y cells, as detected by RT-qPCR. (D). Upregulation of HERV-W env protein levels in HERV-W env-transfected SH-SY5Y cells, as detected by Western blot analysis. ns, not significant, ** $p < 0.01$.
