# Supplementary material for: HERV-W Env Induces Neuron Pyroptosis via the NLRP3–CASP1–GSDMD Pathway in Recent-Onset Schizophrenia
Source: Int J Mol Sci. 2025 Jan 9;26(2):520. doi: 10.3390/ijms26020520 (PMC11765033; doi:10.3390/ijms26020520)
Supplement: Supplementary file 1 [file ijms-26-00520-s001.zip › Supplementary Figure S2.pdf]

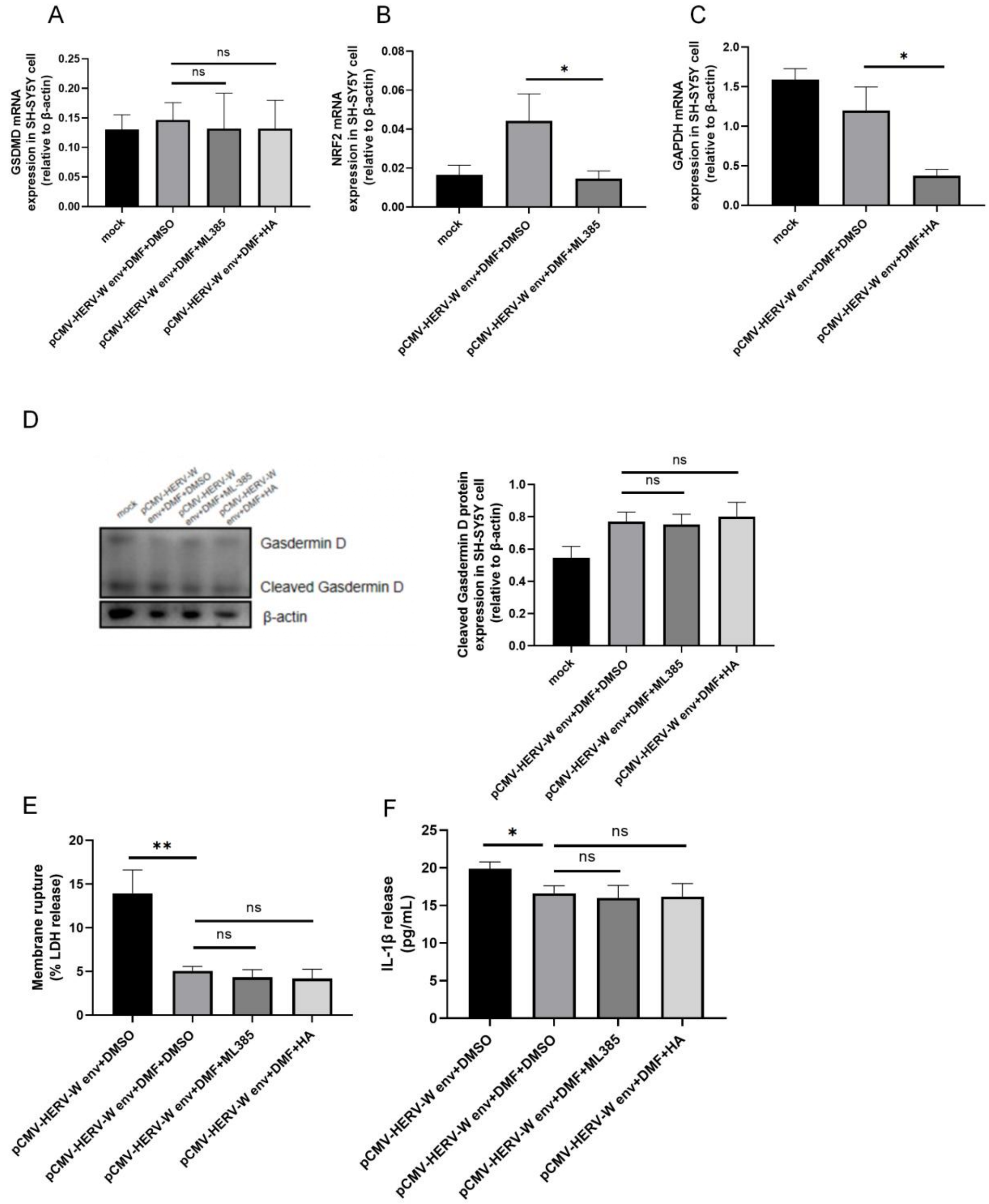

Supplementary Figure S2. The effect of DMF on NRF2 and GAPDH would not affect the inhibition on GSDMD and pyroptosis . (A). ML385 and HA had no effect on the mRNA levels of *GSDMD* in HERV-W env-transfected SH-SY5Y cells with the treatment of DMF, as detected by RT-qPCR. (B). The inhibition of ML385 on the mRNA levels of *NRF2* in HERV-W env-transfected SH-SY5Y cells with the treatment of DMF, as detected by RT-qPCR. (C) The inhibition of HA on the mRNA levels of *GAPDH* in HERV-W env-transfected SH-SY5Y cells with the treatment of DMF, as detected by RT-qPCR. (D). ML385 and HA had no effect on the protein levels of Gasdermin D in HERV-W

env-transfected SH-SY5Y cells with the treatment of DMF, as detected by Western blot analysis. (E). Lack of effect of ML385 and HA on LDH release in HERV-W env-transfected SH-SY5Y cells with the treatment of DMF, measured using the CytoTox 96 LDH Cytotoxicity Assay Kit according to the manufacturer's protocol. (F). Lack of effect of ML385 and HA on IL-1β release in HERV-W env-transfected SH-SY5Y cells with the treatment of DMF, as measured by ELISA. ns, not significant, * $p < 0.05$, ** $p < 0.01$.
