# Supplementary material for: HERV-W Env Induces Neuron Pyroptosis via the NLRP3–CASP1–GSDMD Pathway in Recent-Onset Schizophrenia
Source: Int J Mol Sci. 2025 Jan 9;26(2):520. doi: 10.3390/ijms26020520 (PMC11765033; doi:10.3390/ijms26020520)
Supplement: Supplementary file 1 [file ijms-26-00520-s001.zip › Supplementary Tables.pdf]

Supplementary Tables

Supplementary Table S1. List of primers used in this article

Number	Primer	Forward sequence (5' to 3')	Reverse sequence (5' to 3')	NCBI Gene ID
1	GAPDH	GCTCCTCCTGTTCGACAGTCA	ACCTTCCCCATGGTGTCTGA	2597
2	HERV-W env	CCAATGCATCAGGTGGGTAAC	GAGGTACCACAGACAAAAAATATTCCT	30816
3	CASP1	GAAGAAACACTCTGAGCAAGTC	GATGATGATCACCTTCGGTTTG	834
4	GSDMD	GAGCTTCCACTTCTACGATGCCATG	CCTGCGATCTTTGCCTGTCCTG	79792
5	IL-1β	ATGATGGCTTATTACAGTGGCAA	GTCGGAGATTCGTAGCTGGA	3553
6	GSDME	TGCCTACGGTGTCATTGAGTT	TCTGGCATGTCTATGAATGCAAA	1687
7	NLRP3	GATCTTCGCTGCGATCAACAG	CGTGCATTATCTGAACCCCAC	114548
8	NLRP2	ACGGTGGTGCTGTATGGTC	TCCTCTGCCCAGTCTAGCATT	55655
9	CARD8	ACAAAGGCGATAGATGATGAGGA	AACTGGAACCAAAGTTCAGGG	22900
10	NLRC4	TGCATCATTGAAGGGGAATCTG	GATTGTGCCAGGTATATCCAGG	58484
11	BID	ATGGACCGTAGCATCCCTCC	GTAGGTGCGTAGGTTCTGGT	637
12	NRF2	TCCAGTCAGAAACCAGTGGAT	GAATGTCTGCGCCAAAAGCTG	4780
13	ACTB	CATGTACGTTGCTATCCAGGC	CTCCTTAATGTCACGCACGAT	60

Supplementary Table S2. Antibodies for Western-blot analysis

Antibodies	Source	Identifier	Dilution multiple
CASP1 Rabbit pAb	ABclonal	A16792	1:1000
GSDMD Rabbit pAb	ABclonal	A18281	1:1000
GSDME Rabbit pAb	ABclonal	A7432	1:2000
NLRP3 Rabbit pAb	ABclonal	A12694	1:1000
Syncytin 1 Polyclonal Antibody	Thermo Fisher Scientific	SYCY1-101AP	1:1000
GAPDH Rabbit pAb	ABclonal	AC027	1:5000
β-actin Rabbit pAb	ABclonal	AC006	1:5000

Supplementary Table S3. Comparison of clinical characteristics of serum samples from controls and patients with schizophrenia

Characteristics	Recent-onset schizophrenia (N=20)	Normal control (N=20)
Age (years)	40.83±11.75	43.17±12.75
Education (years)	11.24±3.74	11.06±4.23
BMI (body mass index)	21.54±2.58	20.86±3.47
Gender (F/M)	12/8	9/11
Smokers (N, n%)	8(40%)	5(25%)
